# Characterization of the placental transcriptome through mid to late gestation in the mare

**DOI:** 10.1371/journal.pone.0224497

**Published:** 2019-11-14

**Authors:** Shavahn C. Loux, Pouya Dini, Hossam El-Sheikh Ali, Theodore Kalbfleisch, Barry A. Ball

**Affiliations:** 1 Maxwell H. Gluck Equine Research Center, Department of Veterinary Science, University of Kentucky, Lexington, KY, United States of America; 2 Faculty of Veterinary Medicine, Ghent University, Merelbeke, Belgium; 3 Theriogenology Department, Faculty of Veterinary Medicine, University of Mansoura, Mansoura City, Egypt; Colorado State University, UNITED STATES

## Abstract

The placenta is a dynamic organ which undergoes extensive remodeling throughout pregnancy to support, protect and nourish the developing fetus. Despite the importance of the placenta, very little is known about its gene expression beyond very early pregnancy and post-partum. Therefore, we utilized RNA-sequencing to characterize the transcriptome from the fetal (chorioallantois) and maternal (endometrium) components of the placenta from mares throughout gestation (4, 6, 10, 11 m). Within the endometrium, 47% of genes changed throughout pregnancy, while in the chorioallantois, 29% of genes underwent significant changes in expression. Further bioinformatic analyses of both differentially expressed genes and highly expressed genes help reveal similarities and differences between tissues. Overall, the tissues were more similar than different, with ~ 95% of genes expressed in both tissues, and high similarities between the most highly expressed genes (9/20 conserved), as well as marked similarities between the PANTHER pathways identified. The most highly expressed genes fell under a few broad categories, including endocrine and immune-related transcripts, iron-binding proteins, extracellular matrix proteins, transport proteins and antioxidants. Serine protease inhibitors were particularly abundant, including SERPINA3, 6 and 14, as well as SPINK7 and 9. This paper also demonstrates the ability to effectively separate maternal and fetal components of the placenta, with only a minimal amount of chorioallantoic contamination in the endometrium (~8%). This aspect of equine placentation is a boon for better understanding gestational physiology and allows the horse to be used in areas where a separation of fetal and maternal tissues is essential. Overall, these data represent the first large-scale characterization of placental gene expression in any species and include time points from multiple mid- to late-gestational stages, helping further our understanding of gestational physiology.

## Introduction

Pregnancy is dynamic with a continuous dialog between the conceptus and dam throughout gestation. This dialogue ensures that critical events, such as maternal recognition of pregnancy, establishment of appropriate placentation, angiogenesis, fetal growth and ultimately parturition occur at the proper times and in the proper order to ensure survival of the neonate. Even a small error in early gestation can result in pathologic conditions in later gestation. Despite this, most of the research directed toward the placenta over the past 40 years has focused primarily on either early or late pregnancy, with relatively little understanding of the physiology or gene expression of the mid-gestation placenta.

This lack of mid-gestational studies is a problem not only in the horse, but across all placental mammals. To the best of the authors’ knowledge, there have only been three large-scale gene expression studies in mid-gestation placenta, all performed on microarray platforms in the human or mouse. The human studies examined a range of gestational ages, comparing either first and second trimester placental gene expression[1], or second and third trimester[2]. The remaining study observed placental and embryonic gene expression at E12.5 in the mouse[3]. Other major gene-expression studies have either been performed on abnormal placentae, such as those obtained from cloned pregnancies[4], or on very early or late term placentae[5]. Moreover, no other mid or late gestation study has included the maternal (endometrial) aspect of the placenta. In many species, including human and mouse, it is not possible to separate the maternal and fetal placenta due to hemochorial placentation, making it difficult to study the maternal-fetal interaction during gestation. The horse has epitheliochorial placentation, making it a suitable model for studying the fetal (chorioallantois) and maternal (endometrium) aspects of the placenta, including maternal response.

In the horse specifically, there have been several sequencing and microarray studies conducted during early pregnancy. These studies include fetal membranes and/or endometrium from days eight to sixteen [6–8], oviductal epithelium at day four [9], inner-cell mass and trophectoderm cells [10], induced trophectoderm cells [11], in addition to early (33–34 d) chorionic girdle cells in horses [12], donkeys, mules and hinnies [13–15]. Additionally, our laboratory has examined the kinetics of the C14MC miRNA cluster in pregnancy using a portion of the data presented in this paper [16]. These studies not only identify some of the major changes in gene expression during these time periods, but also highlight the fact that the maternal and fetal aspects of the placenta play very different roles during pregnancy.

Despite the lack of major gene expression studies, placental health is still imperative to maintaining a healthy pregnancy, and the work that has been done on mid-gestation pregnancy has focused on methods of evaluating gestational health. In the horse, these include analyzing various factors in circulation including endocrine markers [17, 18], plasma proteins[19–21] and small RNAs [22]. In women, amniocentesis is frequently used [23–25]; although there is evidence that this may be a useful diagnostic tool in the horse [26], it has not yet been embraced by equine practitioners. Additional equine mid-gestational studies include work on fetal circulation and metabolism[27, 28], steroid production[29–32], fetal fluid composition[33] and placentation [34–36].

In the horse, the initial invasion of the fetal trophoblast into maternal tissues does not begin until 35–42 days of gestation with the formation of the endometrial cups [37], comprising the only form of invasion seen in equine placentation. As the endometrial cups form, the uterine epithelium reforms over the cups and development of microcotyledonary placentation is initiated; these microcotyledons encompass nearly the entire placental surface, but never initiate degradation of the maternal endometrium [38]. Prior to this, myometrial contractions are believed to hold the conceptus at the base of the uterine horn [37]. As placental development continues, the microvilli of the chorioallantois is mirrored by the endometrial sulci to maximize placental surface area and hemotrophic exchange [38], illustrating how the fetal and maternal tissues coordinate to function as a unit. Despite the importance of the endometrium in placentation, it has been largely ignored during earlier research efforts.

Given the overall lack of knowledge about mid-gestational gene expression in the endometrial and trophoblastic placenta, we aimed to characterize the transcriptome of the chorioallantois and the endometrium throughout gestation to gain a better understanding of placental physiology and the complementary roles of fetal and maternal tissues during equine pregnancy. To do so, we utilized next-generation sequencing to evaluate messenger RNA expression at a wide range of gestational time points, including 4 m, 6 m, 10 m and 11 m, employing bioinformatic tools to better understand the kinetics of the placental transcriptome throughout pregnancy.

## Materials and methods

### Animal use and tissue collection

All animal procedures were approved by and completed in accordance with the Institutional Animal Care and Use Committee of the University of Kentucky (Protocols #2014–1215 and 2014–1341). All horses (*Equus caballus*) used in this study were mixed-breed mares ranging from 250 to 600 kg and from four to nine years of age. All animals in this study were bred and owned by the University of Kentucky and mares were housed on pasture with free-choice grass hay available at all times.

Mares were bred via pasture mating, with pregnancy confirmed by transrectal ultrasonography between 14 to 35 days of gestation. Gestational age was determined by the size and morphology of the conceptus during the first ultrasound examination. Paired chorioallantois (CA) and endometrium (EN) were collected post-mortem at gestational ages 4 m, 6 m, 10 m and 11 m, with n = 4 animals per time point.

Following euthanasia, the intact uterus was removed, and full thickness sections of the uterus and placenta were taken from the body of the uterus, approximately 10 cm from the cervix. Gentle traction was applied to separate the chorioallantois from the endometrium manually, and the endometrium was carefully dissected from the underlying myometrium and stroma. Sections of all isolated tissues were stored in RNAlater (Thermo Fisher Scientific, Waltham, MA, USA), with samples held at 4°C for 24 hours, then frozen at -80°C until use.

Additional sections of uterus and chorioallantois were fixed in formalin for 24 hours at 4°C, then transferred to methanol until embedded in paraffin. Following affixation to slides, sections were stained with hematoxylin and eosin using an automated Sakura Prisma slide stainer (Torrance, CA, USA), following manufacturer’s instructions.

### RNA isolation and sequencing

Isolation of RNA from tissue was performed using RNeasy Mini Kit (Qiagen, Gaithersburg, MD, USA), per manufacturer’s instructions. After extraction, RNA was analyzed by NanoDrop® (Thermo Fisher Scientific) and Bioanalyzer® (Agilent, Santa Clara, CA, USA) to evaluate concentration, purity and integrity. All samples had a 230/260 ratio > 1.8, a 260/280 ratio > 2.0 and an RNA integrity number > 8.0.

Library preparation was performed using the TruSeq Stranded mRNA Sample Prep Kit (Illumina), per manufacturer’s instructions. The adapter for Read 1 was AGATCGGAAGAGCACACGTCTGAACTCCAGTCACNNNNNNATCTCGTATGCCGTCTTCTGCTTG, with NNNNNN signifying the index sequence. The read 2 adapter was AGATCGGAAGAGCGTCGTGTAGGGAAAGAGTGTAGATCTCGGTGGTCGCCGTATCAT. All reads were quantified with qPCR. Sequencing was performed on a HiSeq 4000 (Illumina) using a HiSeq 4000 sequencing kit version 1, generating 150 bp paired-end reads (University of Illinois Roy J. Carver Biotechnology Center). FASTQ files were generated and demultiplexed using bcl2fastq v2.17.1.14 Conversion Software (Illumina). All sequencing data have been deposited in NCBI Sequence Read Archive via the Gene Expression Omnibus and are available through GEO Series accession numbers GSE136691 and GSE108279.

### RNASeq data analysis

The sequencing results were trimmed for adapters and quality using TrimGalore Version 0.4.4 (Babraham Bioinformatics; www.bioinformatics.babraham.ac.uk), then mapped to EquCab3.0 [39] using STAR-2.5.2b (github.com/alexdobin/STAR) [40]. Cufflinks-2.2.1 (cole-trapnell-lab.github.io/cufflinks/) [41] was used to quantify data in fragments per kilobase/million (FPKM), with the Equus_caballus_Ensembl_95 gtf file used for annotation (-G).

### Database construction and statistical analyses

For all data analyses, separate databases were created for chorioallantoic and endometrial samples. To qualify for inclusion, genes needed to have an average FPKM > 1 in at least one stage of gestation, with 50% or more samples showing expression. Differentially expressed genes were determined within tissue. Principal components analysis was performed on all genes included in either database to verify clustering. Two samples were identified as outliers (CA_10m_2, EN_4m_2) using K nearest neighbors by gestational tissue and age; these were excluded from all further analyses.

Initial identification of differentially expressed genes was performed using one-way ANOVA, with the Benjamini-Hochberg correction for false discovery rate (FDR P < 0.05). Gene expression as measured by FPKM was compared across gestational ages. Secondary analysis was by gestational age within tissue using the same methodology, directly comparing one gestational age to another.

To assess correlation between tissues, all genes from the chorioallantois and endometrium databases were considered, totaling 13,259 genes. Correlation was assessed using pairwise correlation by gestational age. K-means clustering was used to analyze normalized gene expression patterns across gestation for all DEG. Normalization was performed by gene, with the highest expression set to 1. For each gestational age, mean expression (FPKM) was determined, and this mean was used to normalize gene expression across gestational ages setting the maxima as one. The highly expressed genes were identified by averaging the FPKM for each gestational age, with the maximum of these used to determine order of expression. All statistical analyses were performed in JMP (SAS Institute, version 14.0.0) unless otherwise stated. Descriptive statistics are expressed as mean ± SE.

### Weighted Gene Co-expression Network Analysis (WGCNA)

To better categorize and assess the kinetics of the transcriptome across gestation, we utilized the open-source statistical software tool “R” (http://www.r-project.org). The R-based WGCNA package was used to construct a scale-free network and co-expression modules based on the FPKM data for each gene with an FPKM >1, as defined above[42]. Initially, we determined the mean connectivity (K) for all transcripts and used this data to determine the lowest soft threshold power which still reached a scale-free topology index of 0.90. Using the determined soft threshold power, transcripts were clustered into highly interconnected modules using hierarchical clustering based on topological overlap measures. Each module was assigned a color for ease of reference. The eigengene (the first principal component) from each module was calculated, and Pearson’s correlation coefficient was used to correlate the eigengene to gestational age and fetal gender. Each module significantly correlated with gestational age was extracted and further characterized using Panther DB GO biological process complete (www.pantherdb.org) [43].

Ultimately, chorioallantois and endometrium databases as identified above were evaluated separately to better identify changes occurring across gestation as opposed to across tissue. A soft threshold power of 10 was identified as optimal for chorioallantois, where a soft threshold value of 8 was identified and used for endometrium.

## Results

### Sequencing Profile

Sequencing produced 17.60 ± 1.47 x 10^6^ reads per chorioallantois sample and 19.64 ± 1.55 x 10^6^ reads per endometrial sample. On average, 0.32% ± 0.006% of base pairs did not meet the quality requirements and were subsequently trimmed. Mapping resulted in 94.87 ± 0.05% of reads being successfully mapped to the genome (EquCab3.0). Consequently, the chorioallantois had an average of 15.99 ± 1.43 x 10^6^ uniquely mapped reads per sample, while the endometrium had 18.19 ± 1.51 x 10^6^ uniquely mapped reads per sample.

### Differential gene expression

In total, 12,526 genes were evaluated for differential expression in the endometrium. Of these, 5,932 (47.4%; FDR P-value < 0.05) changed across gestational age. A similar number of genes were evaluated in the chorioallantois (12,615); however, only 3,667 (29.1%) were found to change significantly across gestational age (S1 Table).

**Fig 1:**
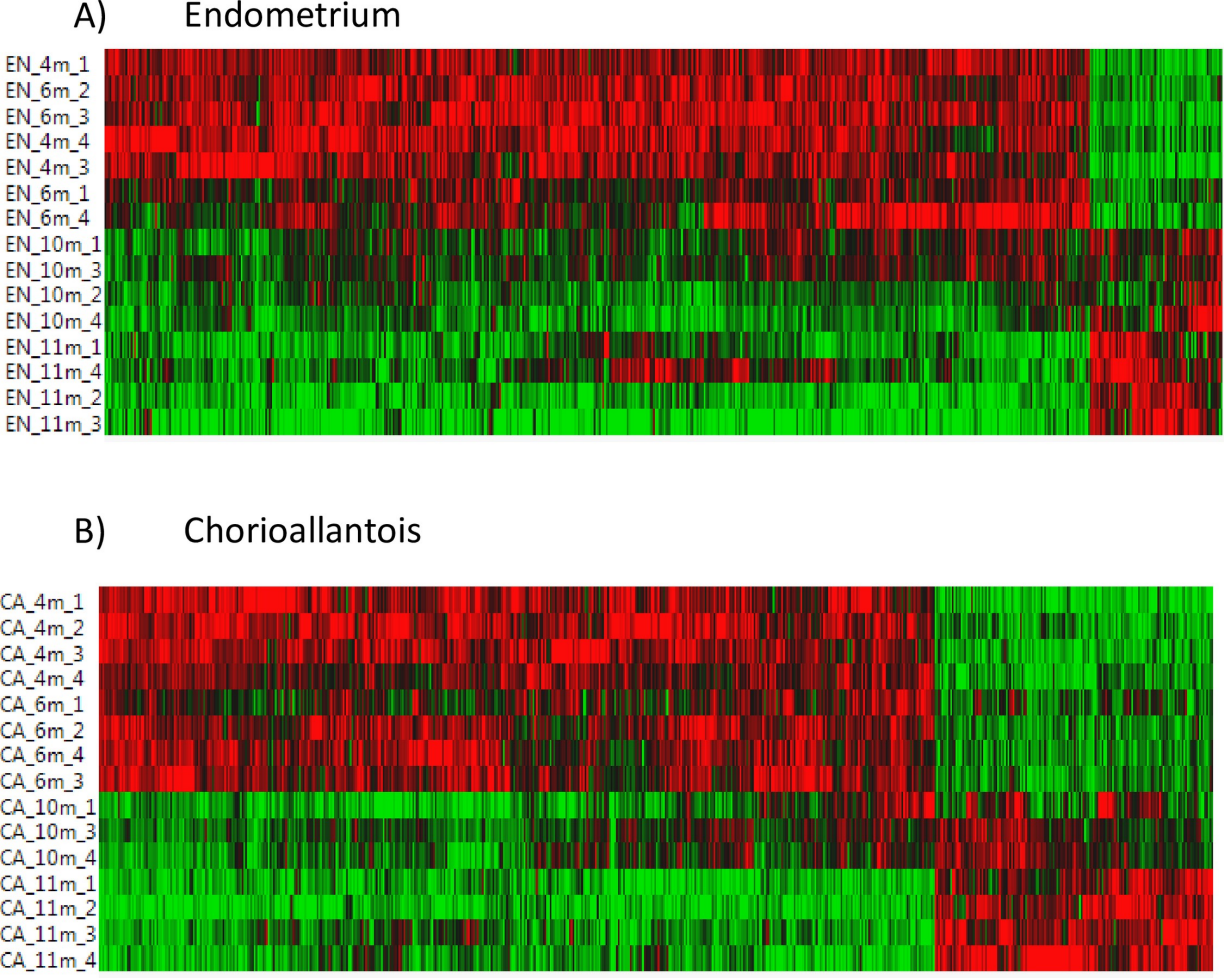
Heat map of endometrium and chorioallantois. Clustered heat map showing relative gene expression / sample for all differentially expressed genes (FDR P-Value < 0.05) at each gestational age. Includes A) endometrium, excluding diestrus and B) chorioallantois at 4, 6, 10 and 11 m gestation.

**Fig 2:**
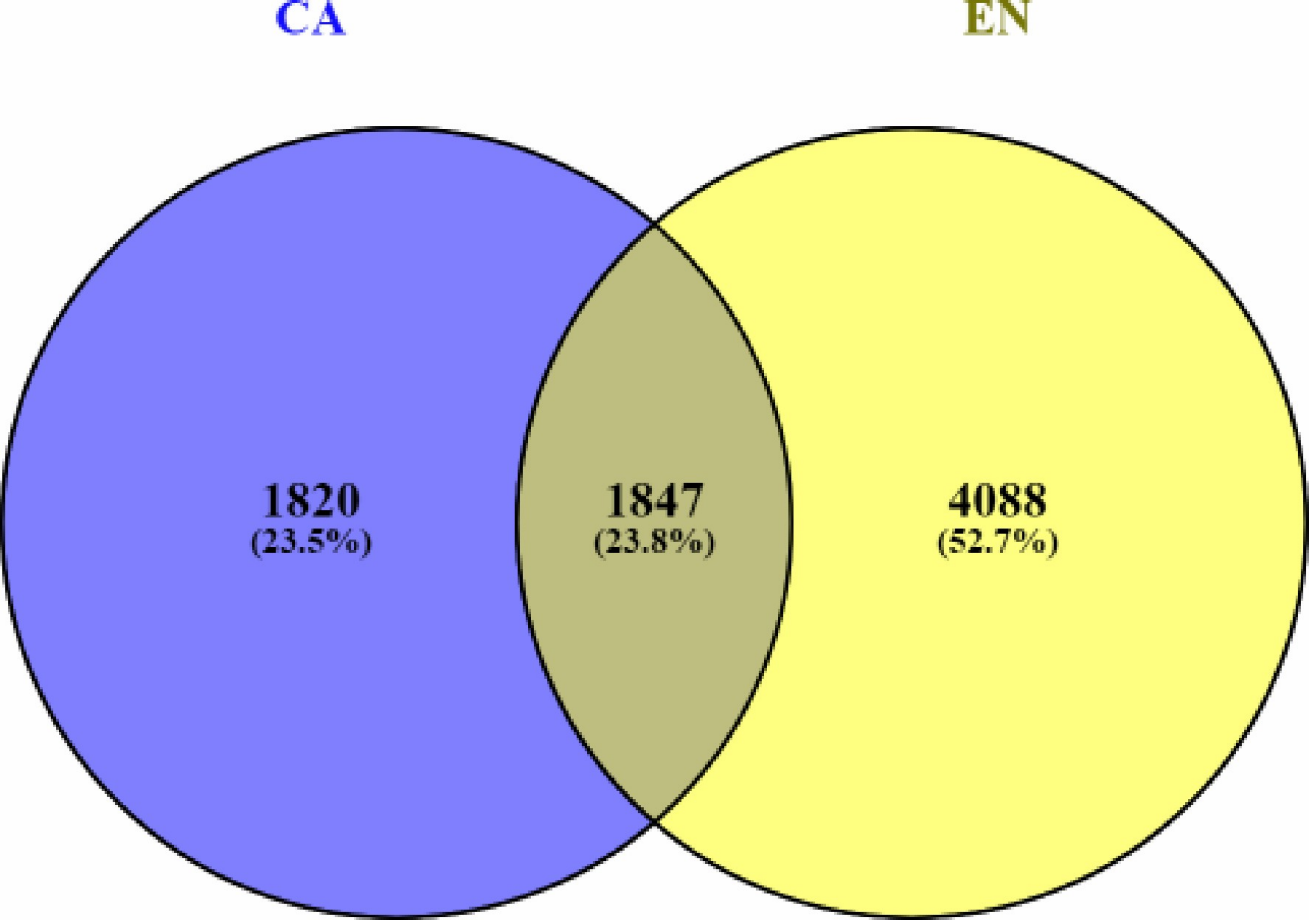
Venn diagram of differentially expressed genes. Graphical representation of the overlap of differentially expressed genes by group (FDR P-Value < 0.05). Includes genes which were differentially expressed in chorioallantois (CA), and endometrium (EN) across gestation.

Expression patterns of differentially expressed genes across gestational ages in the chorioallantois and endometrium were visualized via heat map (Fig 1). Comparing differentially expressed genes between tissues, approximately 25% of genes (1,847) were differentially expressed in both chorioallantois and endometrium (Fig 2).

Most genes (11,881) were expressed in both tissues (FPKM > 1 at 1 or more gestational stages), indicating that the genes which are expressed are similar between the chorioallantois and endometrium, or potentially that there is some level of cross-contamination between the two tissues. Even so, there were 644 genes exclusively included in the EN database (5.68%), while 734 were exclusive to CA (5.54%).

**Fig 3:**
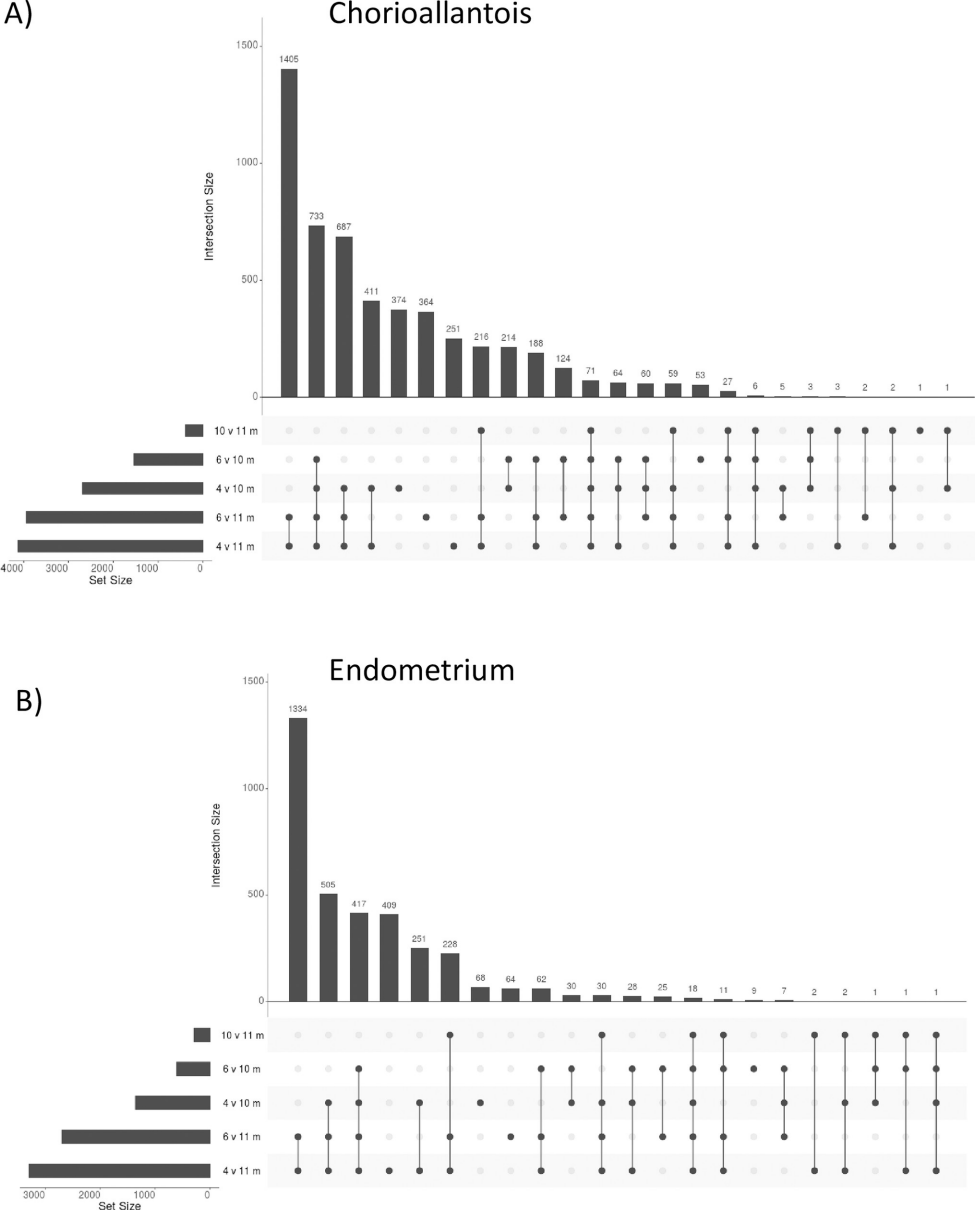
Differential gene expression across gestation. Horizontal bars indicate number of differentially expressed genes per comparison. Vertical bars indicate number of differentially expressed genes shared across timepoints as indicated by black dots; connections indicate two or more conserved time points per transcript.

When comparing gestational stages directly, the largest number of DEG were identified while comparing 4- and 11 m tissues, within either the CA or EN (Fig 3). The only gestational ages which did not have genes changing between them were 4 and 6 m gestation; true for both CA and EN. Despite the similarity in the patterns of DEG, the actual genes changing were quite different between tissues; only 14.2% of DEG were conserved between CA and EN at a given gestational age (range 3.1% - 29.8%).

**Fig 4:**
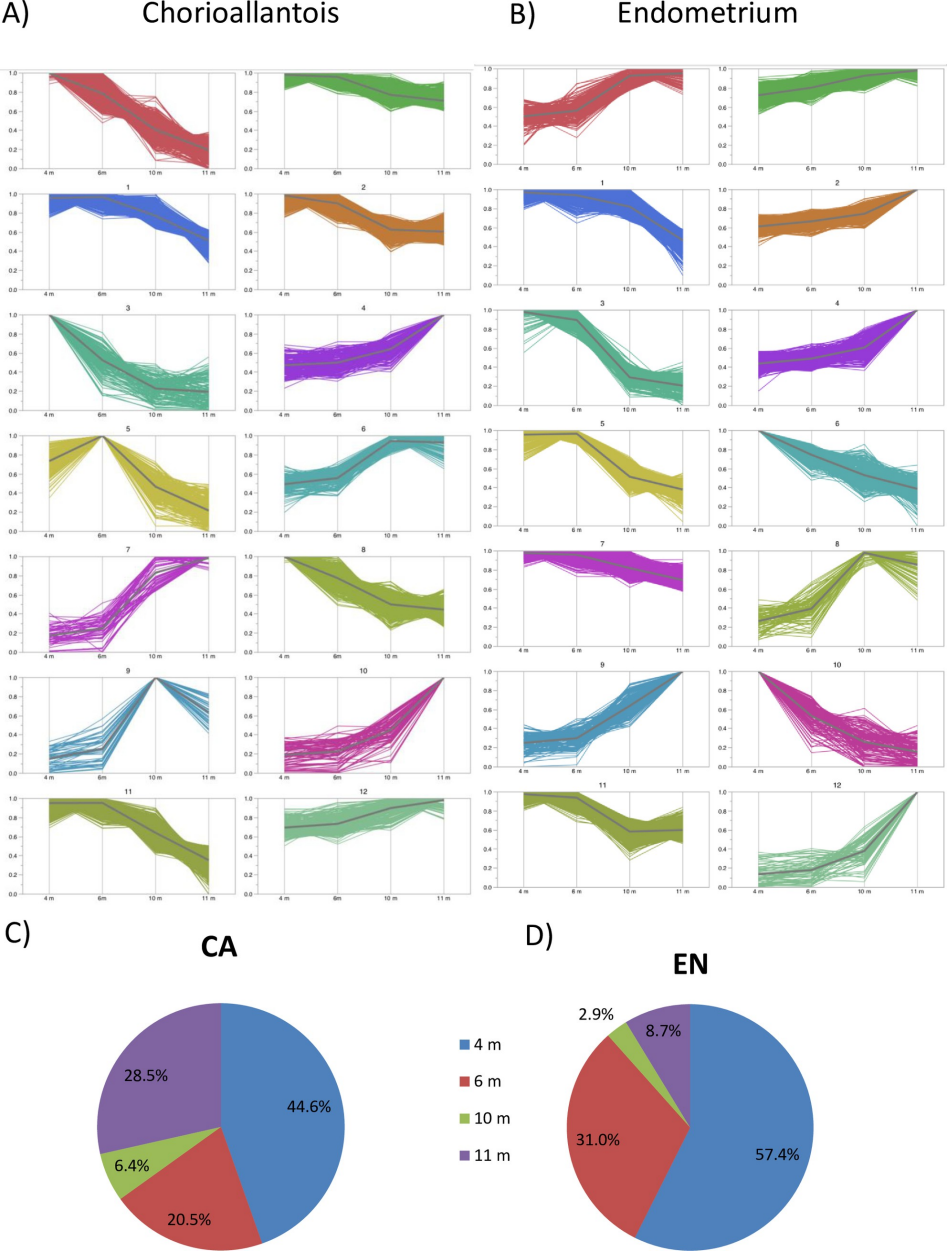
Differential gene expression across gestation. Gene clustering by K-means cluster based on normalized gene expression of genes with an FDR P Value < 0.05 across gestation. Genes were normalized on an individual basis, with the highest expression set to 1. The 14 clusters for A) chorioallantois and B) endometrium are visualized. Pie charts indicate the proportion of genes which are highest at each time point for C) chorioallantois (CA) and D) endometrium (EN). For a more in depth analysis of these data, including gene identities / cluster, please see S2 Table.

When analyzing patterns of normalized gene expression across gestation, the majority of DEG had maximum expression at 4 m gestation (57.4% and 44.6% for EN and CA, respectively; Fig 4C and 4D). Overall, DEG patterns trended towards higher expression at either 4 m or 11 m, with incremental expression decreases across the other gestational ages, as seen in both chorioallantois and endometrium (Fig 4A and 4B). Specific genes present in each cluster are presented in S2 Table.

### Correlation of mRNA and principal components analysis

Despite the similarity of genes in each tissue, directly comparing the CA and EN expression of all genes with an FPKM > 1 resulted in a significant (P < 0.0001), but weak correlation (r = 0.25). Analysis of correlation between CA and EN by month of gestation (Table 1) revealed that all time points were significant (P < 0.0001), with the 4 and 6 m EN and CA samples showing the highest correlation within tissue (r = 0.98 and 0.96, respectively; Table 1). Unsurprisingly, the intra-tissue correlations were consistently higher than the inter-tissue correlations.

**Table 1:** Correlation of tissues by gestational age.

	CA_4m	CA_6m	CA_10m	CA_11m	EN_4m	EN_6m	EN_10m	EN_11m
CA_4m	1.000	0.956	0.769	0.780	0.310	0.368	0.163	0.235
CA_6m	0.956	1.000	0.846	0.768	0.290	0.360	0.160	0.212
CA_10m	0.769	0.846	1.000	0.871	0.313	0.411	0.163	0.171
CA_11m	0.780	0.768	0.871	1.000	0.371	0.454	0.186	0.263
EN_4m	0.310	0.290	0.313	0.371	1.000	0.982	0.465	0.474
EN_6m	0.368	0.360	0.411	0.454	0.982	1.000	0.528	0.476
EN_10m	0.163	0.160	0.163	0.186	0.465	0.528	1.000	0.751
EN_11m	0.235	0.212	0.171	0.263	0.464	0.476	0.751	1.000

Correlation (r) of gene expression (FPKM > 1) between tissues (chorioallantois–CA; endometrium–EN) across gestational ages (4, 6, 10, 11 months GA). All correlations were statistically significant (P < 0.0001), with darker shading indicating higher correlation.

**Fig 5:**
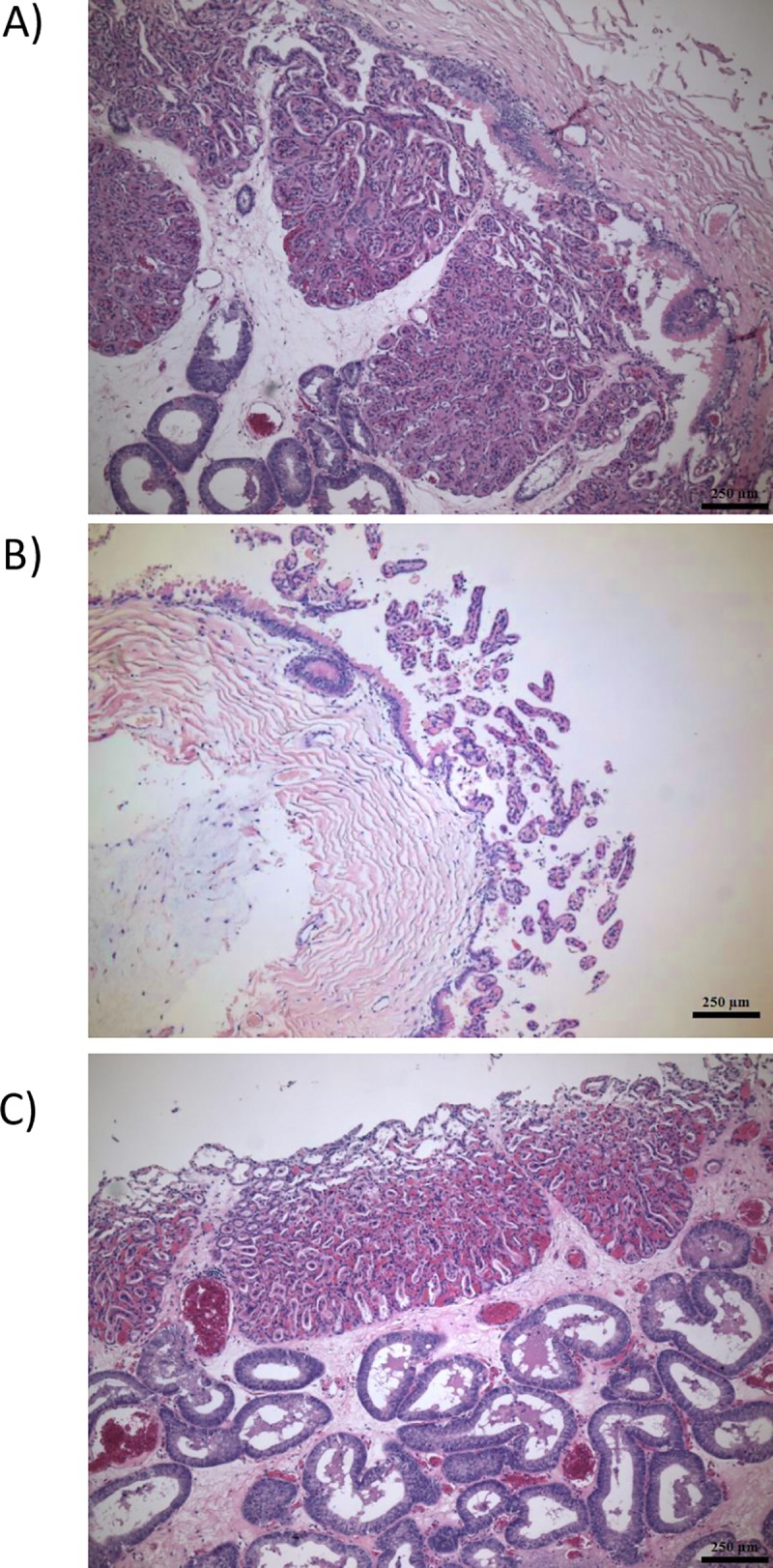
Histological analysis. Evaluation of histological sections stained with H&E in A) intact uterus/chorioallantois; manually separated B) chorioallantois and C) endometrium. EG–endometrial glands; CV–chorionic villi; ALL–allantois. All tissues were derived from a single pregnancy at 10 m gestation.

Part of our motivation to examine the correlation between paired CA and EN samples was to evaluate the level of potential cross-contamination between CA and EN. From our histological analysis (Fig 5), we know that a portion of the chorioallantoic villi remain embedded in the endometrial tissue; however, the exact level of chorionic contribution to the endometrial transcriptome is not clear.

To quantitate the relative contamination of endometrial samples with chorionic microvilli, we examined a Y-chromosome specific gene, eukaryotic translation initiation factor 2 subunit 3, Y-linked-like (*EIF2S3Y*). This gene is Y-specific in other species; however, it’s currently mapped to chromosome 4 in the horse, allowing us to identify gene expression in our current genome without mapping to the Y-chromosome. Out of 8 CA samples from male fetuses, all had moderate gene expression, ranging from a FPKM of 1.25 to 4.64 (3.22 ± 0.47). No expression of *EIF2S3Y* was noted in any of the eight CA samples from female fetuses. Of the endometrial samples, expression in samples associated with a male fetus were considerably lower than in CA (0.17 ± 0.08). Endometrial samples from pregnancies with a female fetus were less likely to have expression of *EIF2S3Y*, although 2/8 had low expression (0.07 ± 0.06 FPKM), a level which is likely to not represent actual transcript data. Overall, endometrial expression of *EIF2S3Y* associated with pregnancies with a male fetus averaged 7.74% of that of the paired chorioallantoic expression.

**Fig 6:**
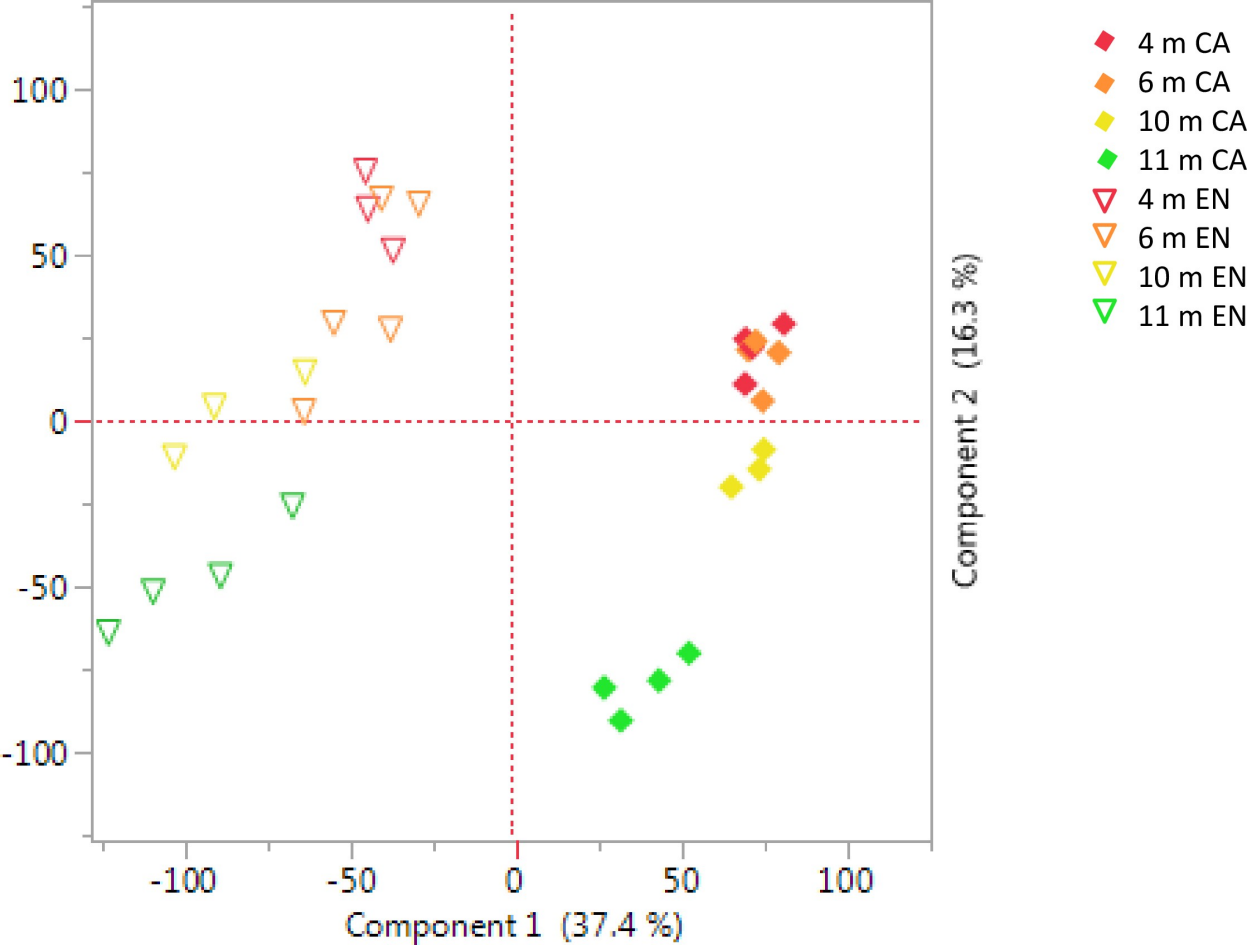
Principal components analysis. Evaluation of clustering of samples by principal components analysis to identify outliers. Diamonds–chorioallantois (CA); Triangles–endometrium (EN). Red– 4 m gestation; orange– 6 m gestation; yellow– 10 m gestation; green– 11 m gestation.

A principal components analysis was performed to evaluate how well the individual samples and gestational ages clustered together (Fig 6). Overall, chorioallantois clustered separately from endometrium, with time points clustering by gestational age. In both tissues, the 11 month samples were the most distinct. Unsurprisingly, the separation by tissue accounts for a larger degree of variation than the separation by gestational age.

### Gene ontology analysis

#### Differentially expressed genes

PANTHER GO-Slim Biological Process identified 53 and 115 pathways using the differentially expressed genes in CA and EN, respectively (S3 Table). Overall, pathways were highly conserved between tissues, with 43 pathways present in both tissues. The conserved pathways were consistent with high rates of cell division, including cell cycle, cellular component organization, DNA repair, intracellular protein transport and metabolic process. Of interest, most immune response pathways were underrepresented in both tissues, including B-cell activation, leukocyte and lymphocyte activation and general immune system processes. Unique endometrial pathways included numerous metabolic processes, including carbohydrate, amino acid, ncRNA, tRNA, DNA and cellular protein metabolic processes. The chorioallantois had fewer unique pathways; these included glycerolipid and glycerophosopholipid metabolic processes, protein polyubiquitination and transmembrane receptor protein tyrosine kinase signaling pathways.

#### Highly expressed genes

Although differential gene expression is the standard for identifying important genes in a dataset, the most highly expressed genes also tell an important part of the story when evaluating tissue function. Therefore, in addition to the analysis of differentially expressed genes, the 250 most highly expressed genes in EN and CA were identified and evaluated, excluding genes lacking annotation (17 and 44 in CA and EN, respectively). Using the PANTHER Overrepresentation Test (Go biological process complete), 274 overrepresented pathways in CA were identified, as well as 195 overrepresented pathways in EN (S4 Table). When comparing the overrepresented pathways between tissues, all but 27 of the EN pathways were also identified in CA; these included coat protein complex I (COPI)-coated vesicle budding, antibacterial humoral response and blood vessel morphogenesis. Pathways identified with high confidence in both tissues include both endocrine and immune-related pathways (estradiol secretion, androgen catabolic process, interleukin-7 related pathways).

A closer examination of the 20 most highly expressed genes / tissue, we revealed that many of the transcripts which have been classically associated with pregnancy and placentation in the horse, including relaxin (*RLN*), aromatase (*CYP19A1*), *EEF1A1* and uteroferrin (*ACP5*; Table 2). Roughly half (9/20) of these transcripts are in the top 20 most highly expressed transcript list for both CA and EN. The list consists of a disproportionately high rate of endocrine-related transcripts (*RLN*, *CYP19A1*, *HSD3B2*, *SPP1*, *PLA2G10*, *INHBA*), immune-related transcripts (*CST3*, *CTSL*, *SERPINA3*, *SERPINA6*, *SERPINA14*, *SPINK7*, *SPINK9*, *LTF*, *S100A6*, *SLPI*), iron-binding proteins (*ACP5*, *FTH1*, *HBA2*, *LCN2*, *SERPINA14*), and serine protease inhibitors (*SERPINA3*, *SERPINA6*, *SERPINA14*, *SPINK7*, *SPINK9*). Other categories include extracellular matrix proteins (*ECM1*, *SPARC*, *MMP26*), transport proteins (*ACP5*, *GM2A*, *HBA2*, *LCN2*), and antioxidants (*PRDX1*, *SOD3*).

**Table 2:** Most abundant transcripts in chorioallantois (CA) and endometrium (EN).

Abbr.	Full Name	CA rank	EN rank	Function (s)
**ACP5**	uteroferrin; acid-resistant phosphatase 5	9	4	iron binding, transport protein
**APOE**	apolipoprotein E	11	36	lipid transport; innate and adaptive immune response
**CST3**	cystain C	54	16	innate immune system (antimicrobial function)
**CTSL**	cathepsin L	5	15	proteinase (substrates include collagen, elastin, alpha-1, protease inhibitor)
**CYP19A1**	aromatase	3	13	endocrine
**ECM1**	extracellular matrix protein 1	16	254	extracellular matrix protein
**EEF1A1**	eukaryotic translation elongation factor 1 alpha 1	18	24	translation (delivers tRNA to ribosome)
**FABP1**	fatty acid binding protein 1	10	28	binds hydrophobic ligands, including long-chain fatty acids
**FTH1**	ferratin heavy chain 1	57	18	iron binding
**GM2A**	GM2 ganglioside activator	6	19	transport protein (glycolipids)
**HBA2**	hemoglobin subunit alpha 2	19	98	iron binding
**HSD3B2**	HSD3 beta steroid delta-isomerase 2	12	41	endocrine
**INHBA**	inhibin subunit beta A	110	10	endocrine
**LCN2**	lipocalin 2	8	5	transport protein (small, hydrophobic molecules); endocrine; immune response; iron binding
**LTF**	lactotransferrin	155	6	iron binding; immune response (innate)
**MMP26**	matrix metallopeptidase 26	68	12	extracellular matrix protein; degrades collagen, fibronectin, fibrinogen, beta-casein; activates MMP9
**PLA2G10**	phospholipase A2 group X	15	138	endocrine (hydrolyzes glycerophospholipids to produce free fatty acids)
**PRDX1**	peroxiredoxin 1	20	47	antioxidant; immune response (antiviral activity of CD8(+) T cells)
**RLN**	relaxin	1	9	endocrine
**S100A6**	S100 calcium binding protein A6	14	32	calcium binding; immune response; stimulates hormone release
**SERPINA3**	serpin family A member 3	353	20	immune regulation; target of NR4A1
**SERPINA6**	serpin family A member 6	112	3	immune regulation; corticosteroid-binding protein (major transport protein for glucocorticoids and progestins in blood)
**SERPINA14**	serpin family A member 14	4	2	iron binding (in conjunction with uteroferrin); immune response
**SLPI**	secretory leukocyte peptidase inhibitor	7	7	protects tissues from serine proteases (affinity for trypsin, elastase and cathepsin G), immune response
**SOD3**	superoxide dismutase 3	50	17	antioxidant
**SPARC**	secreted protein acidic and cysteine rich; osteonectin	17	33	extracellular matrix-associated protein; binding protein (calcium, copper, etc)
**SPINK7**	serine protease inhibitor, kazal type 7	90	14	immune regulation
**SPINK9**	serine protease inhibitor, kazal type 9	2	1	immune regulation
**SPP1**	secreted phosphoprotein; osteopontin	13	89	endocrine; immune response (acts as cytokine)
**STC1**	stanniocalcin 1	77	11	calcium, phosphate-regulating hormone; estrogen and progesterone responsive; early pregnancy marker (implantation)
**WFDC2**	WAP Four-disulfide core domain 2	28	8	protease inhibitor

Rank refers to relative abundance / tissue; rank 1 is transcript with the highest average abundance across gestation. Abundance determined by averaging all samples by time point (n = 4), then using the highest average value across gestation for ranking.

### Weighted Gene Co-expression Network Analysis (WGCNA)

#### Chorioallantois

**Fig 7:**
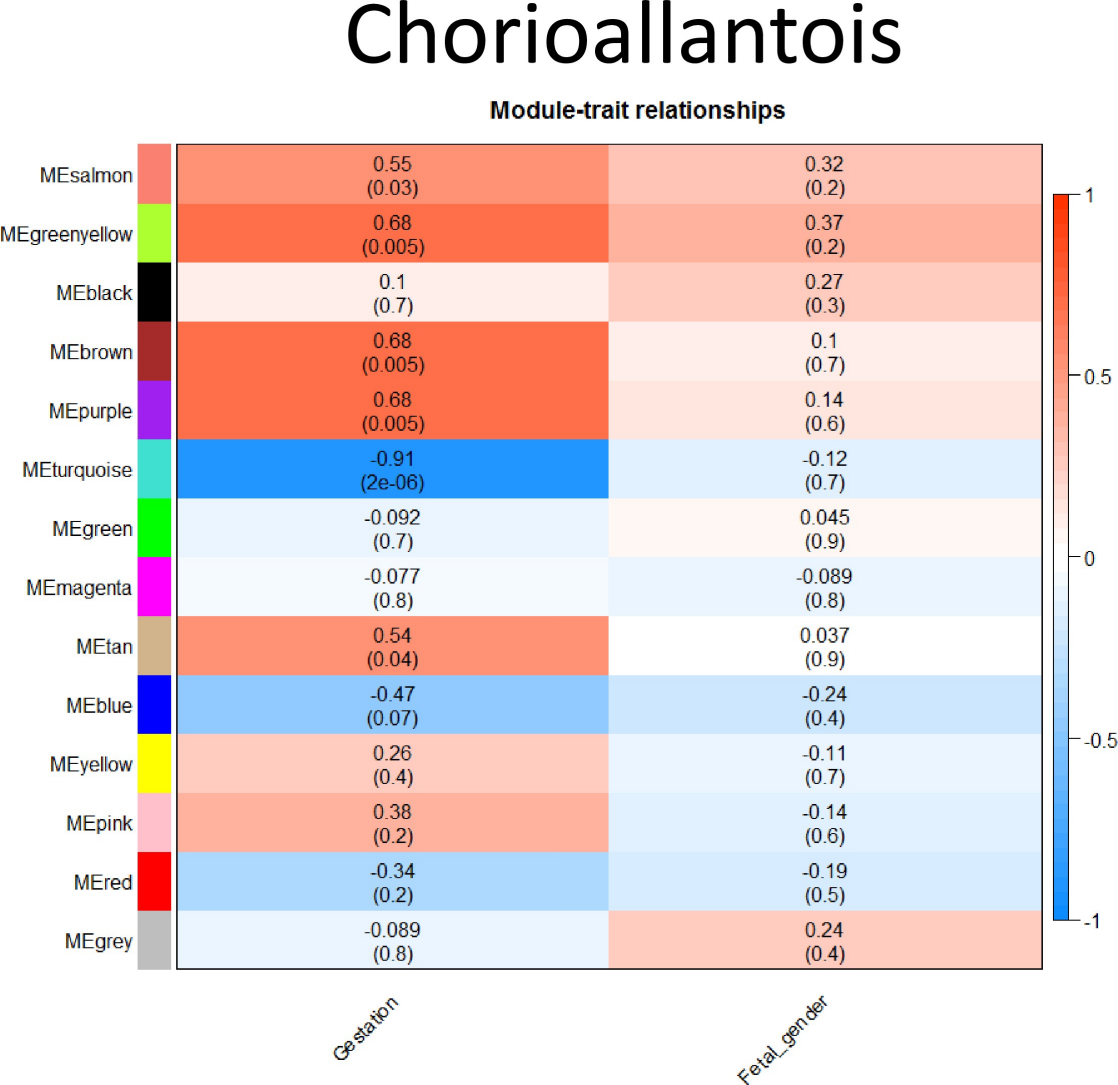
Weighted Gene Co-Expression Network Analysis (WGCNA) in chorioallantois. Correlation (r) of each module identified by WGCNA to external factors (Gestation and fetal gender). Color and depth of color corresponds to depth of correlation, with positive correlation indicated in red, negative correlation indicated in blue. Significance (P-value) of each module to each external factor presented in parentheses ().

In total, 14 modules were identified within chorioallantois; six of which were significantly correlated to gestational age (Fig 7). None of the modules were significantly correlated with fetal gender. Gene identification was extracted from all significant modules and evaluated via GO biological process complete (S5 Table). The one negative module (turquoise) was associated with GO terms including regulation of protein exit from endoplasmic reticulum, mitotic spindle organization, phosphatidylinositol phosphorylation and histone lysine methylation. Only two modules with a positive correlation to gestational age had significant pathways identified; these included the brown and purple modules. The other modules had no overrepresented pathways identified. The brown module included pathways related to peptidyl-proline hydroxylation, collagen fibril organization, cartilage development, regulation of ossification and negative regulation of immune system process. The purple module was more immune-specific, with pathways including defense response to virus, immune effector process and response to biotic stimulus.

#### Endometrium

**Fig 8:**
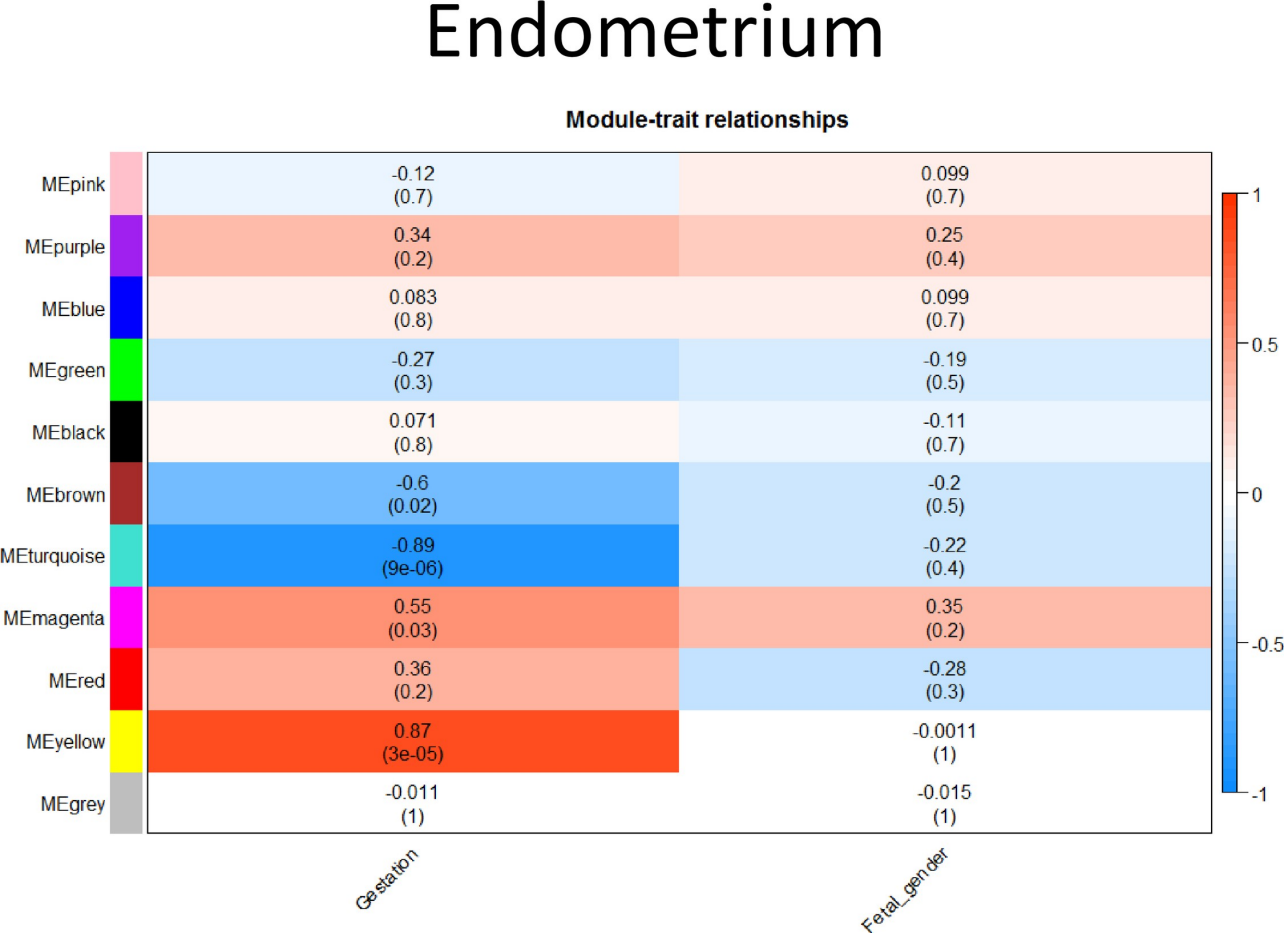
Weighted Gene Co-Expression Network Analysis (WGCNA) in endometrium. Correlation (r) of each module identified by WGCNA to external factors (Gestation and fetal gender). Color and depth of color corresponds to depth of correlation, with positive correlation indicated in red, negative correlation indicated in blue. Significance (P-value) of each module to each external factor presented in parentheses ().

Eleven modules were identified in the endometrium, four of which were significantly correlated with gestational age (Fig 8). Two of these had a negative correlation, whereas the other two had a positive correlation with gestation. Like the chorioallantois, no module was significantly correlated with fetal gender.

Positively correlated modules had a number of overrepresented pathways, including spindle assembly, mitochondrial transport, microtubule-based transport, and RNA modifications, as well as numerous metabolic processes (S6 Table). Modules with a negative correlation had overrepresented pathways identified associated with cytoplasmic translation, organ morphogenesis, limb development and peptide biosynthesis. Other pathways of note included anion transport, metabolic processes and organic substance transport.

To better compare the kinetics of specific transcripts in the CA and in the EN, all transcripts present in a module significantly correlated with gestational age were identified. No transcript was present in more than a single module per tissue. In total, 11,623 transcripts were present in the 10 significant modules, 4,807 of which were present in both CA and EN (Table 3). Of these, 3,617 (75%) were present in modules with matching correlation (e.g. both CA and EN had negative correlation to gestational age), where the other 1,190 (25%) were present in modules which had positive correlation to gestational age in one tissue, but negative correlation in the other.

**Table 3:** Comparison of WGCNA expression by tissue and module.

Correlation		-0.91	0.54	0.55	0.68	0.68	0.68		
	Module	CA_turquoise	CA_tan	CA_salmon	CA_green_yellow	CA_purple	CA_brown	Unique genes	Total
-0.89	EN_Turquoise	3295	53	36	34	55	831	7180	8189
-0.6	EN_brown	259	1	1	9	3	69	548	631
0.55	EN_magenta	28	0	0	0	0	21	67	88
0.87	EN_yellow	70	1	1	0	0	40	197	239
	Unique genes	1457	17	19	32	33	917		
	Total	5109	72	57	75	91	1878		

Comparison of WGCNA gene module expression between endometrium (EN) and chorioallantois (CA), including correlation of module to gestational age (GA). The number in the table indicates the number of shared transcripts between any two modules. Blue shading indicates negative correlation between modules and gestational age. Red shading indicates positive correlation between modules and gestational age, where gray shading indicates one module has a positive correlation with gestation, while the second module has a negative correlation with gestation. Unique genes are genes present in only one module with significant correlation to gestational age.

## Discussion

This study represents the first report based on next-generation sequencing to examine gene expression serially within the chorioallantois and the endometrium throughout mammalian gestation. As such, it provides a more complete picture of the function of each of these tissues, serving as a reference to better understand gestational physiology not only in the mare, but in other species as well.

Overall, there was a large degree of transcriptional crossover between the two tissues creating the fetal/maternal interface, with upwards of 90% of transcripts present in both the CA and EN database; however, the correlation between these shared transcripts was weak (r = 0.25). Similarly, in the WGCNA module data (Table 3), nearly 25% of transcripts were present in modules with opposing correlation, showing these transcripts increase during gestation in one tissue while decreasing during gestation in the other.

The similarity in identified transcripts combined with low expression correlation may be partly explained by the degree of chorionic villi retention within the endometrial tissue following separation of the chorioallantois from the endometrium. Adding to the complexity, the degree of contribution of each transcript will vary based on multiple factors including expressing cell type, cellular makeup, and degree of microcotyledonary retention. To estimate the contribution of chorionic tissue in endometrial samples, we examined the expression of *EIF2S3Y*, a male-specific transcript. For this transcript, it appears that the endometrial expression is approximately 8% of that seen within the chorioallantois. Again, this level is likely to vary significantly; however, it provides a starting point in estimating chorionic contribution to endometrium given the equine microcotyledonary placentation. Additional evidence supporting the minimal contribution of chorioallantoic tissue in the endometrium is the number of transcripts which increase throughout gestation in one tissue while decreasing in the other. For example, 3-beta hydroxysteroid dehydrogenase 2 (HSD3B2) and solute carrier organic anion transporter family member 2A1 (SLCO2A1) both increase throughout gestation in CA while decreasing in EN, while ATP-binding cassette transporter (ABCA1) and nuclear receptor subfamily 4 group A member 2 (NR4A2) exhibit the opposite pattern. The level of contamination present is not likely sufficient to mask the true expression patterns between the tissues.

Another important consideration when evaluating this dataset is that these data were generated using whole tissue samples. Both chorioallantois and endometrial tissues are comprised of a diverse population of cells, including, but not limited to trophectoderm, endothelial cells, glandular epithelium, stromal cells and immune cells. Although these transcripts can be localized at a tissue level, it is not currently possible to localize transcripts at a cellular level on a large scale without performing single-cell sequencing. This becomes important particularly in instances of immune cell migration; for example, in humans, leukocytes comprise up to 32% of cells found in first trimester decidua [44]. Horses have a similar influx of leukocytes into the endometrium while the endometrial cups are present (roughly 35–120 d gestation) [45], although the number, type and localization of these leukocytes varying considerably during this time [46, 47]. Although care was taken to avoid the area proximal to the endometrial cups, leukocytes may still have been elevated in the 4 month samples, although this elevation was not apparent in the 4 m histology samples (data not shown).

Initial evaluation and classification of genes of interest based on gene ontology was done through Panther [43], showing most overrepresented pathways in both tissues were ones traditionally associated with cell division, transcript processing and protein production (S3 Table). This is consistent with the rapid growth known to occur during gestation. The prominence of endocrine-associated factors and pathways in the chorioallantois should not come as a surprise; it’s role in steroidogenesis is very well established [48]. However, the role of the endometrium in gestational endocrinology has only been studied minimally.

In the horse, aromatase has previously been reported in the non-invasive trophoblast during early [49], and mid to late gestation [50]. Although aromatase activity was detected in mid-gestation endometrial tissues, this activity was considerably lower than that in the chorioallantois and hypothesized to occur in the fetal tissues remaining in the endometrium [50]. Although we cannot rule this out completely, transcript expression levels suggest that this the endometrium itself is an important source of aromatase transcript, as the transcript expression patterns are different between the two tissues and the levels of endometrial transcript are higher than would be expected for contamination alone (24 ± 16% of CA expression).

Relaxin was found to be highly expressed in both the chorioallantois and endometrium (Table 2), despite being previously described as specific to the chorioallantois [51]. Production of relaxin in pregnancy is known to vary from species to species, including the endometrium (pigs) [52]. Although perhaps best known for its role in relaxing the pelvic ligaments prior to parturition, relaxin also has a number of additional functions, including angiogenesis, uterine and vaginal growth and inhibition of myometrial contractility during early gestation (reviewed in [53]). The high concentrations of relaxin transcript are intriguing and suggest relaxin is integral for the maintenance and support of equine pregnancy throughout gestation.

The other prominent steroidogenic enzyme present in the endometrium was HSD3B2. Previously known as HSD3B1, this enzyme has been reported in the equine chorioallantois [32] and testis [54], as well as early pregnant porcine endometrium[55], but hasn’t previously been studied in the equine endometrium. In contrast, INHBA has been previously reported to be endometrium-specific [56], although these data suggest that it is also expressed at a lower, yet still significant, level in the chorioallantois (Table 2).

Additional knowledge comes from where the two tissues differ. For example, two of the top three EN-specific pathways were related to vesicle budding. The existence and importance of placenta-derived vesicles is well established; believed to be important for facilitating feto-maternal communication, these vesicles are present in maternal circulation as well as fetal fluids [57–59]. That said, they are hypothesized to have a strictly fetal origin [59], making the EN-exclusivity of this pathway worth further examination. These vesicles could also deliver histotroph, the uterine secretions which help support and maintain pregnancy.

The role of the both tissues in the immune system is highlighted as well, as many of the top 20 most highly expressed genes are immune-related (Table 2), with a number of immune-related pathways identified in both tissues (S3 and S4 Tables). The most highly expressed gene overall was *SPINK9*, a serine protease inhibitor which was the most abundant transcript in EN, as well as the second most highly expressed transcript in CA (Table 2). Research into SPINK9 has primarily been in skin, with no known role in pregnancy [60]. In skin, SPINK9 has been shown to inhibit kallikrein-related peptidases (KLKs), particularly KLK5, as it is able to fully thwart KLK5’s ability to degrade fibrinogen [60]. Moreover, SPINK9 functions as an antimicrobial peptide which is able to kill multiple strains of Escherichia coli [61], and likely helps protect the placenta from bacterial invasion.

Many of the most highly expressed transcripts result in proteins which were identified in our previous work characterizing the proteome of fetal fluids [26] and the cervical mucus plug [62]. This confirms the importance of many of these products to pregnancy in the horse, as well as suggesting that some of these products could be produced in the placenta then transported to surrounding fluids and structures to help sustain the pregnancy. Analyzing data via WGCNA allowed the identification of genes which change synchronously throughout gestation, providing a more careful evaluation of the pathways being altered through gestation. By first identifying modules with consistent expression patterns, then identifying which modules change significantly through gestation, confidence in pathway identification can be increased. Additional confidence can be put into pathways which are overexpressed in two or more significant modules (S5 and S6 Tables).

This work represents the first serial study of the chorioallantois and endometrium through mid- to late-gestation based upon next-generation sequencing. These data highlight the dynamic changes occurring in these tissues throughout gestation, as well as providing information on the individual and combined function of the placental tissues. Although a number of pathways and molecules were highlighted in this manuscript, we could not hope to thoroughly describe all of the changes occurring, and as such, we sincerely hope that researchers will delve further into these data, using them to better understand their specific niche of gestational physiology.

## Supporting information

S1 Table P-Values, FDR P-Values, and locus for all genes in chorioallantois (CA) and endometrium (EN) throughout gestation. Significance calculated using a one-way ANOVA including all gestational stages (4 m, 6 m, 10 m, 11 m).
(XLSX)

S2 Table Gene clustering by K-means cluster based on normalized gene expression of genes with an FDR P Value < 0.05 across gestation. Genes were normalized on an individual basis, with the highest expression set to 1. Identity of the genes / cluster / tissue, including normalized value are included.
(XLSX)

S3 Table Pathways identified for differentially expressed genes (FDR P-value < 0.05) in chorioallantois and endometrium PANTHER GO-Slim Biological Process. In comparison tab, over represented pathways are highlighted in green; under-represented pathways in red.
(XLSX)

S4 Table Pathways identified for 250 most highly expressed genes in endometrium and chorioallantois using PANTHER GO biological process complete. Includes direct comparison of pathways identified in each tissue.
(XLSX)

S5 Table WGCNA analysis for chorioallantois. Each tab represents a separate module, including module correlation to gestational age and associated P-value, the list of genes included in the module and pathways identified by PANTHER Go Biological Process Complete for the genes included in each module (if applicable)
(XLSX)

S6 Table WGCNA analysis for endometrium. Each tab represents a separate module, including module correlation to gestational age and associated P-value, the list of genes included in the module and pathways identified by PANTHER Go Biological Process Complete for the genes included in each module (if applicable)
(XLSX)
